# The status of pediatric surgery in Malawi: a narrative mini-review

**DOI:** 10.3389/fped.2023.1195691

**Published:** 2023-07-06

**Authors:** Celina Flocks Monaghan, Chiara Pittalis, Elaine Byrne, Israa Hussein, Tiyamike Chilunjika, Bip Nandi, Eric Borgstein, Jakub Gajewski

**Affiliations:** ^1^ Institute of Global Surgery, School of Population Health, Royal College of Surgeons in Ireland, Dublin, Ireland; ^2^ Centre for Positive Health Sciences, Royal College of Surgeons in Ireland, Dublin, Ireland; ^3^ School of Medicine, Royal College of Surgeons in Ireland, Dublin, Ireland; ^4^ Department of Surgery, University of Malawi College of Medicine, Zomba, Malawi; ^5^ Department of Surgery, Kamuzu Central Hospital, Lilongwe, Malawi; ^6^ Michael E. Debakey Department of Surgery, Baylor College of Medicine, Houston, TX, United States; ^7^ Centre for Global Surgery, University of Stellenbosch, Cape Town, South Africa

**Keywords:** pediatric, surgery, Malawi, global surgery, NSOAP, sub-Saharan Africa

## Abstract

**Introduction:**

Pediatric surgery is essential to a well-functioning health system. Unmet surgical needs contribute to 6.7% of pediatric deaths in Malawi. Understanding the current state of pediatric surgical care in Malawi is necessary to recognize gaps and opportunities in service delivery and to develop evidence-based national planning and solutions.

**Methods:**

This narrative mini review synthesized the literature on the state of pediatric surgery in Malawi through the pillars of the World Health Organization's Health System Building Blocks. A search of PubMed, Embase, and Scopus databases was executed to identify relevant studies and a thematic analysis was performed. Further, to ensure contextual accuracy, pediatric surgeons from Malawi were consulted and involved in this review.

**Results:**

Twenty-six papers were identified. In Malawi's central hospitals, there are six specialist pediatric surgeons for a pediatric population of more than 8 million. There is limited pediatric surgical capacity at the district hospitals. There is little to no written evidence of the national governing and finance structures in place for pediatric surgical services.

**Discussion:**

In countries like Malawi, where a significant portion of the population comprises children, it is crucial to recognize that pediatric services are currently inadequate and fall short of the required standards. It is crucial to prioritize the enhancement of services specifically designed for this age group. This review aims to shed light on the existing gaps within pediatric surgical services in Malawi, providing valuable insights that can inform the development of comprehensive national surgical planning strategies.

## Introduction

Approximately 1.1 billion children and adolescents, predominantly from lower-middle income countries (LMIC), live without access to safe surgical care (1). The 2015 Lancet Commission on Global Surgery (LCoGS) report on the state of surgical care worldwide highlighted these critical inequalities in surgical delivery (1, 2). The report and subsequent calls to action from the LCoGS emphasized the need for a systematic National Surgical, Obstetric and Anesthesia Planning (NSOAP) approach to be globally adopted (2, 3). The LCoGS guidelines do not stipulate specific targets for pediatric surgery, despite recognizing the importance of improving access for this population. In response to this gap, the Global Initiative for Children's Surgery (GICS) advocated that understanding existing gaps in pediatric surgical services is imperative for ensuring that universal health coverage encompasses the healthcare needs of all (4). The GICS proposed a list of “Optimal Resources for Children's Surgery” that detail what surgical services should be required and provided at every level of healthcare (4).

Currently, six countries in sub-Saharan Africa (SSA) have fully developed NSOAPs, Zambia, Nigeria, Madagascar, Rwanda and Tanzania (5). Nigeria was the first of them to incorporate pediatric surgical care into its plan using the GICS recommendations and a pediatric modified WHO assessment tool (6). In 2019, the Southern African Development Community (SADC) committed to developing NSOAPs for all 14 of their member states (7). However only three member states: Zambia, Zimbabwe, and Tanzania, have accomplished this, none including pediatric specific targets (5).

Malawi, a SADC member state, has not yet developed a NSOAP, despite the commitment. Inclusion and investment in pediatric surgical care is imperative for the future of Malawi, as children under 14 years old' (around 8.24 million) account for nearly half of the nation's population (8). The risk of major economic impact from disability due to untreated surgical conditions is threatening (9). There are an estimated two million children in Malawi who may have an incapacitating, but surgically treatable condition needing management (9). A 2020 household study in Malawi, found untreated surgical conditions contributed to 6.7% of all pediatric deaths in the country (10). To address this need, evidence-based development of an NSOAP must be conducted. However, there is no current synthesis of this evidence. Therefore, the aim of this review is to take stock of the current state of pediatric surgery in Malawi, to provide such information for future surgical capacity development.

## Methods

**Figure 1:**
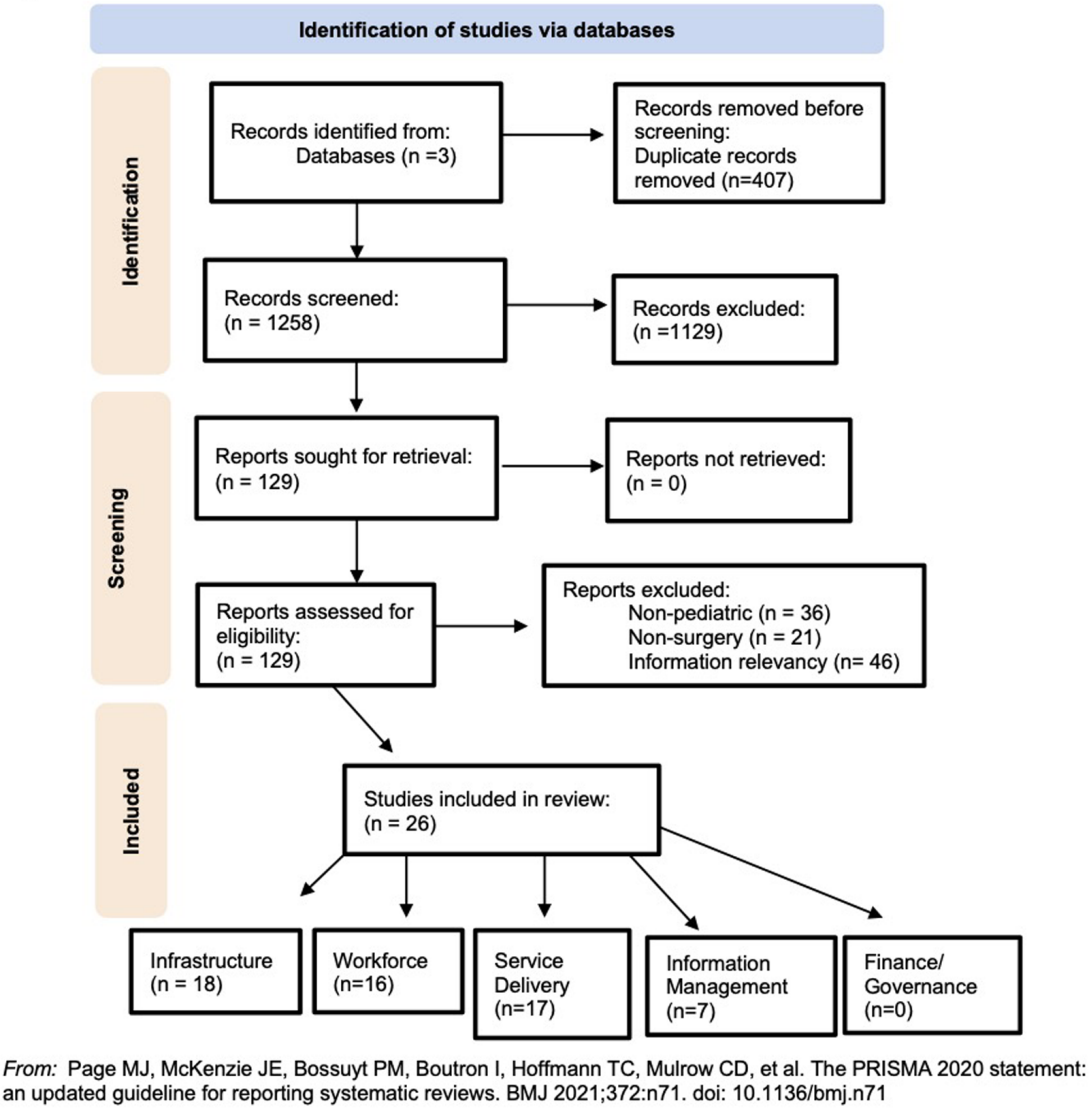
The PRISMA flow diagram for the narrative review detailing the database searches, the number of abstracts screened, and the full texts retrieved.

This narrative mini review on pediatric surgery in Malawi was conducted in accordance with a systematic review procedure described by Petticrew and Roberts (11) and is reported using the Preferred Reporting Items for Systematic Reviews and Meta-Analyses (PRISMA) guidelines (12) (Figure 1). The search strategy was developed in collaboration with a medical librarian at the Royal College of Surgeons in Ireland. An electronic search was conducted in PubMed National Library of Medicine, Embase, and Scopus databases. The search encompassed the following search string, (((“pediatric surgery” OR “paediatric surgery”) AND Malawi) OR ((child* OR infant* OR neonate*) AND (surgery OR surgical)) AND Malawi), adjusted to each database. Papers in English from 2010 to March 2023 were included to ensure the most current publications were retrieved.

The initial search was conducted on December 6, 2022, and an updated search was conducted on June 1, 2023. Identified articles were downloaded, and titles and abstracts were screened by two authors (CFM, IH) using the online systematic review platform Rayyan (Rayyan Systems Inc., Cambridge, MA, USA). During this phase, the inclusion criteria were established to include studies encompassing any aspect of pediatric or child surgery, particularly focused on surgical care provision, studies discussing the availability or lack of surgical services for children in the country, and studies reporting on surgical outcomes and complications in children, this ensured the selection of all pertinent scientific articles. Furthermore, there were no restrictions imposed on the type of publication or research design. Conversely, studies conducted outside Malawi or not involving Malawian patients, along with articles that did not report on surgical care or conditions, were excluded. Any conflicts that arose were resolved by a third reviewer (CP). Subsequently, a full-text review of the selected articles was conducted. The included articles focused on surgical care provided to children, documented or discussed the availability or lack of surgical services for children in the country, or reported on surgical outcomes and complications in children.

Data was extracted from eligible studies using Microsoft Excel. This review utilized indicators from the NSOAP framework which track progress based on the World Health Organizations (WHO) Health Systems Building Blocks as a framework to synthesize the extracted data (2, 3). The NSOAP indicators operationalize the six pillars of infrastructure, workforce, service delivery, information management, finance, and governance (3) tailored to the specifics of surgical care.

To ensure the accuracy and relevance of the study, experts in pediatric surgical care from Malawi were involved at all stages of this review. These experienced professionals validated the findings from the literature and provided valuable insights into the current practices and challenges in pediatric surgery provision.

## Results

The included screened articles consisted of quantitative studies, qualitative studies, mixed-method studies, and secondary reviews, which all aimed to assess or describe system elements of surgical care in Malawi (Figure 1). Twenty-six articles with information relevant to at least one NSOAP-defined building block were included in this review. Several papers included in this review contain information pertaining to more than one NSOAP building block. Consequently, these papers have been utilized in multiple sections of the analysis, to effectively capture the multidimensional nature of the literature. A summary of these findings can be seen in Table 1. This section reports only on what has been published in the literature included in this review.

**Table 1:** Summary of findings.

NSOAP pillar	Strengths	Gaps
Infrastructure	• Two public central referral hospitals with pediatric specialty care, with one pediatric specific surgical and intensive care unit. • One private specialty pediatric orthopedic hospital. • Established electronic consultation network between district hospitals and pediatric surgeons giving guidance on treatment and referral needs.	• Unnecessary referrals lead to overburden on central hospitals. • Frequent unavailability of pediatric sized supplies both surgical and anesthetic. • Lack of diagnostic imaging options leads to surgery as means of diagnostic.
Workforce	• Increase in available pediatric surgeons in recent years, from 1 surgeon in 2010 to 6 surgeons by 2022. • Future workforce improvements with COSECSA pediatric specialty trainees. • Task-shifting of minor surgeries to clinical officers at district level gives greater access.	• Number of surgeons does not meet WHO recommendations. • Hesitancy from doctors who do not specialize in pediatric care and clinical officers to perform operations on children, leading to higher rates of referrals.
Service Delivery	• District hospitals have capacity to treat burns, minor injuries, and some congenital anomalies. • Central hospitals treat more complex burns, congenital anomalies, cancers, neurological conditions, and ophthalmological conditions.	• High rates of non-operative treatment in facilities with non-pediatric specialty providers, either referring or sending home for non-operative care.
Information Management	• Central hospitals track pediatric surgical procedures when able to electronically or in logbooks. • District hospitals encouraged to keep track of procedures electronically or in logbooks.	• Governmental HMIS does not record all pediatric surgical procedures. • Issues with follow up and data accuracy. • District hospital surgical data are difficult to keep track of.
Financing	• No clear information found in literature regarding budget allocation or financing systems in place for pediatric surgical care.	
Governance	• No definite information found in literature regarding governance and governmental oversight in pediatric surgical care.	

### Infrastructure

Eighteen articles included evidence of infrastructure, such as surgical facilities, supplies, and referral systems. Malawi's healthcare system has three tiers linked by a referral system, primary care centers, secondary district hospitals, and tertiary central hospitals (13). Surgical services are only available at the secondary district and tertiary central hospital levels (9, 13). District hospitals, located in 25 of Malawi's districts have basic surgical capacity, and ability to provide regional, spinal, ketamine and general anesthesia for surgical procedures (13, 14). However, pediatric size supplies/equipment are commonly unavailable in district hospitals (15). A 2020 study found equipment such as pediatrIc oropharyngeal airway and endotracheal tubes, necessary for administering anesthesia, to be most frequently unavailable (15).

Malawi has four central referral hospitals: Kamuzu Central Hospital (KCH), Queen Elizabeth Central Hospital (QECH), Zomba Central Hospital, and Mzuzu Central Hospital. The literature indicates that KCH and QECH are the facilities serving as referral centers for pediatric cases. There is no data on what infrastructure Mzuzu and Zomba Hospitals have for pediatric surgical services. KCH, located in the capital Lilongwe serves the central region of Malawi (13, 16). The main operating theater at KCH has four fully functional operating rooms that can perform pediatric surgeries (16, 17). There is no separate pediatric intensive care unit at KCH for perioperative and postoperative services (18). The only pediatric intensive care unit in Malawi is the Mercy James Centre for Pediatric Surgery and Intensive Care (MJC), located within the grounds of QECH in Blantyre (19, 20). Opened in 2017, MJC has six pediatric ICU beds, three pediatric operating theaters, and a 60-bed pediatric surgical ward (20). QECH itself is equipped with seven theaters capable of conducting pediatric surgeries and has pediatric diagnostic imaging modalities including ultrasonography, CT, and x-ray (18, 21–23). Due to the specialty care capacity, MJC and QECH are the major referral centers within the country for pediatric cases needing advanced surgical treatments (19, 21). There are also private hospital facilities, located in central areas, that conduct specialized pediatric surgical services. The Beit CURE International Hospital in Blantyre, provides orthopedic, plastic, and reconstructive surgeries for pediatric patients (24, 25).

The referral system in Malawi allows for transfer of more complex surgical cases which cannot be managed locally from district hospitals to central hospitals (26). However, several studies reported that the inefficiency and inappropriateness of these referrals led to higher risk of over-triage and delays in surgical intervention for pediatric patients (27–29).

### Workforce

Sixteen articles contained evidence regarding the surgical workforce, including allied health providers providing surgical treatments. In 2010, there was only one pediatric surgeon serving a population of 5.98 million pediatric patients (30). By 2022, there were four pediatric general surgeons (9, 24, 31) and two pediatric neurosurgeons (32).

There are no specialist surgeons in the district hospitals, which instead rely on means of task shifting so that surgical interventions are conducted by clinical officers and general medical officers (14, 33). While there is evidence that clinical officers in district hospitals surgically treat children, there is no data on the number who perform these surgeries and the volume at which they do so (33, 34). As suggested by Maine et al., the tendency for district hospitals to transfer pediatric cases highlights clinical officers' limited skills to care for children who may need surgical procedures, regardless of their complexity (27).

Task-shifting also occurs at central hospitals. The literature reports that when there are no pediatric surgeons, operations are conducted by general surgeons, medical doctors, or clinical officers, and are assisted by general anesthesiologists who do not specialize in pediatric care (17, 28, 35, 36). In these facilities, clinical officers perform minor burn surgeries, foreign body removals, and ventriculoperitoneal (VP) shunt placement (35). Further, general surgeries and urology cases are more often performed by medical doctors (29, 34, 35). However, these non-specialty providers have varying confidence levels operating on children (35). One study reported that among facilities where there was no surgeon available, there was a tendency to operate on less complicated, adult patients, while pediatric or more complex cases were referred to the central hospitals or may be sent home for non-operative care (28). A lack of pediatric care training, pediatric anesthesia providers, post-operative critical care, and follow-up abilities contribute to this reluctance (28, 29).

### Service delivery

Seventeen articles comprised information regarding service delivery, including surgical volume at hospitals, system coordination, and safety. There is no comprehensive nationwide data on the number of surgeries performed on children in Malawi. In a review of records from 2011 to 2019 at KCH, 342 pediatric patients underwent burn operations including split-thickness skin graft, debridement, escharotomy, and amputation (13, 37). In a case review conducted between February 2012 and October 2015, KCH had 1680 pediatric surgical admissions and consultations of congenital colorectal disease (22). Of which, 82 pediatric patients were admitted with anorectal malformations, and 26 underwent surgical intervention (22). These operations included exploratory laparotomy, diverting colostomy, posterior sagittal anorectoplasty, and anal dilation (22). In patients with Hirschsprung's Disease, 41 operations performed, included exploratory laparotomy, rectal biopsy, and definitive pull-through (22). Review of MJC admissions from its opening in 2017 until 2019, showed 5,205 outpatient visits, 3,730 theater cases, 3,249 pediatric ward admissions and 579 PICU admissions (19, 20). Pediatric neurosurgeries and treatments of traumatic brain injuries are conducted at QECH and KCH (32, 38). A retrospective analysis report from the Beit CURE International Hospital (BCIH) found that from 2012 to 2013, a total of 1,154 pediatric orthopedic operations were conducted in all public and private facilities in Malawi (24). 53% of these cases (*n* = 609) were performed at BCIH (24); the most common pathologies treated were clubfoot, genu valgus, and burn contracture (24). Staff from BCIH are also periodically seconded to Mzuzu Central Hospital and operate on some 45–75 children per year at that facility (24, 25).

In central hospitals, the absence of perioperative imaging support contributes to the higher rates of non-specific admission diagnoses and misdiagnoses, leading to a reliance on operative intervention as a means of diagnosis (17). In pediatric operations performed by clinical officers and other general surgeons, Reid et Al. reported concerns that a lack of training could lead to a risk of infection (18). This was further exemplified in a study from KCH in 2019, showing that clinical officers, not neurosurgeons, performed shunt operations for hydrocephalus treatment (18). However, in these cases of task-sharing, there is no distinct variability in mortality and complication rates between clinical officers and medical doctors (18, 34, 35).

Pediatric patients needing surgical treatment account for the majority of trauma operations performed at the district level (33). Evidence from district level hospitals show that among pediatric procedures, the most performed operations are hydroceles, hernia repairs, male circumcision, and clubfoot repairs (14, 33). Some of these facilities also reported to have the capacity to surgically treat cleft lip and imperforate anus (14). While some congenital anomalies can be treated at the district level, patients below the age of 15 account for most referrals from district hospitals and other central hospitals (21).

### Information management

Seven articles contained data on information management. There is no evidence in the literature that the Malawian Ministry of Health's Health Management Information System (HMIS) tracks pediatric surgery information and there is no national mandate to ensure record and accuracy of this data (24). Individual hospitals are responsible for tracking pediatric surgical patient information (39). These data are meant to be sent from the facilities to an HMIS officer at the district office to be entered into the district health information system (39). However, multiple studies in this review reported issues with the health record systems, including incomplete data and information tracking for pediatric patients (16, 21, 35). District and central hospitals keep surgical logbooks to track procedures; some in electronic databases and some in handwritten paper clinical logbooks (39). Statistics regarding the number of admissions, number of operations and common diagnoses are provided upon request to the Central Monitoring and Evaluation Division at the Ministry of Health (MOH) (39). In central hospitals, there are surgical patient databases, which an HMIS clerk is meant to use to record final diagnosis, length of stay, treatment summaries, and indicate if a surgical procedure was performed (17, 22, 39). However, a 2014 study at KCH found that 50% of general and congenital pediatric surgery patients in each subspecialty had missing outcome data (35). Further, limitations with the inpatient registry caused records of many pediatric patients, particularly outpatient and short-stay cases, not to be captured in the system (35). An audit of pediatric deaths at KCH also found that some information of patients who died could not be traced in the facilities records and that discharged patient records were also frequently missing (16). Pittalis et al. noted that collection of referral data from district hospitals, which typically includes data on pediatric referrals, is unattainable due to the lack of standard recording systems differentiating surgical patients from medical cases (21).

### Financing

There are no data in the relevant literature on budget allocation or governmental financing support for pediatric surgery in Malawi. Private facilities, namely the Beit CURE International Hospital, charge their adult patients for specialist plastic and orthopedic surgeries, and then utilize those funds to sustain free pediatric surgical services (24, 25).

### Governance

No clear evidence of any governance structure for pediatric surgery exists in the available literature. Only one article mentioned the Malawi Ministry of Health Child Protection and Justice Act 2010, which takes responsibility for preventing premature deaths and disability in children (9).

## Discussion

This review provides a comprehensive analysis of the available evidence regarding pediatric surgical care in Malawi. These findings contribute valuable insights to the existing literature in conjunction with validation from surgical experts. All findings related to each building block, with an exception to the information regarding workforce, were confirmed by the surgical expert team. The information found has direct implications for the improvement of surgical services for pediatric patients in Malawi and may serve as evidence for NSOAP development. Notably, a significant gap in the country's capacity to deliver services pertains to the lack of comprehensive national survey data on pediatric surgical activity. While demand for services is evident, exemplified by an estimated 2.2 million children living with surgically treatable conditions (9), the absence of precise procedure records inhibits the ability to gauge the extent to which this is being met. As a target benchmark, the Lancet Commission on Global Surgery recommends 5,000 surgical procedures per 100,000 population (2). An insufficient national data collection system is a major challenge, as there is no baseline information on how far from recommended targets Malawi may be, with no possibility of drawing meaningful population level conclusions.

The first and second iterations of Malawi's Health Sector Strategic Plan prioritize the provision of reliable, complete, accessible, timely, and consistent health information data to be used for evidence-based decision-making in the health system (40). The tracking of pediatric surgical data has not been included in this priority setting. While the importance of data is noted in the strategic plan the current routine health information system doesn't support the collection and use of pediatric surgical data. Although DHIS2 has been adopted as the national health information system paediatric surgical data is still collected via paper records and is not routinely entered into DHIS2 (39, 41). Therefore, the literature in this review relies on individual hospital reporting, particularly from two central hospitals, KCH and QECH, and the private not-for-profit facilities.

Further, the available evidence shows that with six pediatric surgeons, the demand for pediatric surgical care far exceeds the supply available (9). The current recommended need for a surgical workforce would be 41 pediatric surgeons for the population of 8.24 million children in Malawi (30). However, this reported number of pediatric surgeons from the literature is not the most current. According to the pediatric surgery experts from Malawi on this review team, the current number of pediatric surgeons is five. The fluctuation of these estimates reflects recent provider departures and the retention of newly graduated providers, but absence of a collective database renders this information difficult to find. As a constituent member of the College of Surgeons of East, Central and Southern Africa (COSECSA) Malawi has increased its pediatric surgical workforce and therefore widened access to specialty training in the last decade (14, 42). The availability of a designated pediatric surgical hospital (MJC) and strategic international partnerships for specialty training, has assisted in this success towards scaling up the workforce (19). Evidence from 2021 shows that pediatric surgical trainees assisted in 1,745 surgeries in Malawi (42). Comparatively, Tanzania, also a COSECSA member and a nation that has an NSOAP, has been able to grow its workforce to 12 pediatric surgeons (43). Collectively, the pediatric surgical workforce in the COSECSA region comprises only 52 specialists (44). However, priority for building this specialty workforce has proven to be successful in other regions of sub-Saharan Africa. A notable example is Nigeria, the first country to include pediatric surgery in its NSOAP. As a result, the pediatric surgery workforce in Nigeria has experienced significant growth, expanding from approximately 35 specialists in 2006 to over 130 by 2021 (45). This achievement highlights the positive impact of prioritizing pediatric surgery within the healthcare system and serves as an encouraging model for other countries in the region.

This review underscores the absence of comprehensive guidance regarding the financing and governance of pediatric surgery in Malawi. It is evident that national authorities across Sub-Saharan Africa (SSA) have accorded low priority to funding pediatric surgery, a fact supported by substantial corroborating research (31). Notably, surgical provisions are absent from national funding programs in Malawi, and the government has yet to allocate a dedicated budget for this essential domain (46). Despite incurring substantial costs, public central and district hospitals provide surgical services free of charge to both adults and children (46). Malawi's shortage of specialists creates a reliance on task-shifting of pediatric surgical delivery to clinical officers, mainly at the district level (34, 35). Clinical officer hesitancy due to lack of pediatric training, and financial burdens deter district hospitals from expanding their services and embracing task-shifting responsibilities (34, 35, 46). However, as evidenced in Uganda, the use of task shifting for pediatric care has been seen as a beneficial way of making services more accessible and relieving the burden of the unmet need (47). To mitigate concerns of safety and provider hesitancy, it is imperative that the Ministry of Health (MOH) prioritize financing pre-service and in-service specialty trainings of clinical officers, particularly in administering pediatric anesthesia. Additionally, the implementation of MOH governance of regulations for task shifting of pediatric services should be prioritized.

The findings of this review illustrate the critical nature of these resource deficiencies. The lack of comprehensive procedure records highlights a knowledge gap that necessitates immediate attention and further research to improve health outcomes for children in Malawi. Therefore, it is paramount for the government to actively engage and invest in the sector, ultimately ensuring that the planning and implementation of an NSOAP significantly enhances health outcomes for all segments of the population.
